# Emphysema in active farmer’s lung disease

**DOI:** 10.1371/journal.pone.0178263

**Published:** 2017-06-14

**Authors:** Thibaud Soumagne, Marie-Laure Chardon, Gaël Dournes, Lucie Laurent, Bruno Degano, François Laurent, Jean Charles Dalphin

**Affiliations:** 1 Service de Pneumologie, CHU de Besançon, Besançon, France; 2 Service de Physiologie-Explorations fonctionnelles, CHU de Besançon, Besançon, France; 3 Service de Pneumologie, CHI de Vesoul, Vesoul, France; 4 Service d’Imagerie thoracique et cardiovasculaire, CHU de Bordeaux, Pessac, France; 5 U1045, Université de Bordeaux, Centre de Recherche Cardio-Thoracique, Bordeaux, France; 6 EA 3920, Université de Franche-Comté, Besançon, France; 7 UMR 6249 Chrono-environnement, Université de Franche Comté, Besançon, France; Ohio State University, UNITED STATES

## Abstract

**Background:**

Farmer’s lung (FL) is a common type of hypersensitivity pneumonitis. It is often considered that fibrosis is the most frequent finding in chronic FL. Nevertheless, three cohort studies have suggested that some patients with chronic FL may develop emphysema. We aimed to evaluate the current prevalence of emphysema in active FL, to describe the radiological and functional features of emphysema in active FL, and to identify risk factors associated with emphysema in this population.

**Methods:**

Patients aged over 18 years with active FL were prospectively recruited through the SOPHIA study (CPP Est; P-2009-521), between 2007 and 2015. Each patient had complete medical history screening, clinical examination, high resolution computed tomodensitometry, bronchoalveolar lavage, pulmonary function tests and serum precipitins.

**Results:**

Among 33 patients with active FL, the prevalence of emphysema in this series of incident active FL cases was higher (48.5%) than that of fibrosis (12%) and was not dependent on smoking habits. Most patients with emphysema did not have lung hyperinflation. The possible risk factors for emphysema in active FL were a longer duration of exposure to organic dusts, and at a higher level.

**Conclusion:**

Emphysema is found in half of patients with active FL and may be influenced by exposure patterns.

## Introduction

Hypersensitivity pneumonitis (HP) is an interstitial lung disease caused by an immune response to a variety of antigens to which patients have previously been sensitized. Farmer’s lung (FL) is one of the most common types of HP and its prevalence is estimated to be between 0.2 and 1.5% in farmers [1]. Both acute and subacute FL are most often “active” forms, as they associate ongoing or recurrent respiratory symptoms and lymphocytic alveolitis on bronchoalveolar lavage (BAL) [2, 3]. Chronic FL can either be active or residual, the latter being associated with the disappearance of lymphocytic alveolitis on BAL [2]. It is commonly acknowledged that lung fibrosis is the most frequent finding in chronic HP, either active or residual [4, 5]. However, three cohort studies of FL have suggested that some patients with chronic FL may develop emphysema [6–8]. Nevertheless, these studies date from more than fifteen years ago, and thus, exposure features may have changed over time, and at the same time, imaging techniques have improved. Furthermore, little is known about emphysema in active FL. Indeed, in these studies, no distinction was made between active or residual forms. Few functional features, especially those associated with lung hyperinflation, and no detailed description of emphysema were provided. The recent European Academy of Allergy and Clinical Immunology position paper highlighted this absence of detailed description and emphasized that further research is needed into the factors involved in the development of emphysema in HP [9].

Recently, we reported two cases of active FL with emphysema, including the first description of combined pulmonary fibrosis and emphysema (CPFE) in HP [10, 11]. In the current study, we aimed to: (i) evaluate the prevalence of emphysema in active FL, (ii) describe the radiological and functional features of emphysema in active FL patients, and (iii) identify the risk factors associated with emphysema.

## Materials and methods

### Study design and patients

Patients aged > 18 years with FL were prospectively recruited through the SOPHIA study between 2007 and 2015 [12]. All patients met the criteria for active FL [2], namely: 1) chronic exposure to organic dusts (dairy farming); 2) compatible clinical manifestations; 3) BAL lymphocytosis (> 30% for non- and ex- smokers and >20% for current smokers or < 20% with variegated white blood cell count if BAL was performed within 48 hours following the exposure [13, 14]); and 4) bilateral ground glass and/or mosaic and/or expiratory air trapping and/or poorly defined centrilobular nodular opacities. When the association of high resolution computed tomodensitometry (HRCT) and BAL did not allow the investigators to reach a final diagnosis of HP with confidence or when patients where considered to have residual HP (form in which symptoms related to exposure to an offending antigen and BAL lymphocytosis have disappeared), the patient was not included. In addition to HRCT and BAL, each patient had a complete medical history, clinical examination, pulmonary function tests and serum precipitins. All examinations were performed at the time of the first clinical visit. Ethics committee approval was received from the local Ethics Committee (CPP Est; P-2009-521), and written consent was obtained from all subjects. Two cases included in the present study have been previously reported [10, 11].

### HRCT

HRCT was performed in the department of radiology of the University Hospital of Besançon according to the standardized procedure used for incident cases of interstitial lung disease. We used a single-detector helical CT scanner (GE Hispeed, Milwaukee, Wisconsin, US) with the following protocol: 1.0-mm collimation, 0.8 pitch, 140 kV, 200–280 mA, 1 second [15].

CT readings were performed independently by two thoracic radiologists with more than ten years experience each (FL and GD), and who were blinded to the patient’s clinical data and diagnosis. Disagreements were resolved by consensus. The following findings were examined separately: reticulation, honey-combing, ground glass, traction bronchiectasis, interlobular septal thickening, nodules, micro-nodules, emphysema, mosaic and air trapping, using the definitions of the Fleischner Society glossary of terms [16]. Each specific feature was quantified visually in each lobe by each reader using a semi-quantitative scale from 0 to 4, as the proportion of features occupying the lung parenchymal volume of the lobe (0: no feature; 1: 1–25%; 2: 26–50%; 3:51–75%; 4: more than 75%).

Patients where considered to have an emphysematous form of FL if they had bilateral emphysema in at least 3 lobes according to the American Thoracic Society criteria [17].

Automatic quantification of emphysema was assessed with Myrian software (Intrasense, Montpellier, France) using low attenuation area per cent (LAA%), as previously described [18]. The percentage of LAA% was derived from the voxel frequency distribution histogram and represented voxels less than a threshold value of -950 Hounsfield units. LAA% represents emphysema extent observed on CT scans [19].

### Precipitins tests and BAL

The choice of antigenic panel was based on the recognised causes of FL and HP due to moulds at an international [20, 21] and local level [22, 23]. The antigenic panel tested included *Lichtheimia corymbifera*, *Wallemia sebi*, *Eurotium amstelodami*, *Fusarium oxysporum*, *Saccharopolyspora rectivirgula*, *Thermoactinomyces vulgaris*, *Streptomyces mesophile and Saccharomonospora viridis*. Antigen extracts were produced and tested as described in a previous article [22]. The immunological methods used were agar gel double diffusion and electrosyneresis on cellulose acetate as described previously [23].

BAL was performed using three 50 mL aliquots of a sterile 0.9% saline solution. The cellular composition of the BAL fluid was then determined [24].

### Pulmonary function tests and 6-minute walk test

Routine spirometry, constant-volume body plethysmography and single breath lung transfer for carbon monoxide (TLCO) were performed in accordance with recommended techniques (Platinum Elite; MGC Diagnostics Corporation, Saint Paul, Minnesota, USA) as described elsewhere [25]. Lung hyperinflation was defined as a functional residual capacity above the upper 95^th^ percentile of the predicted values (z-score > 1.64) [26]. A 6-minute walk test (6MWT) was performed as previously described [27]. Exercise-induced hypoxia during 6MWT was defined as a 4% decrease from the baseline saturation and exercise-induced dyspnoea was defined by a Borg scale > 7 after 6MWT.

### Statistical analysis

Agreement between the observers in the assessment of CT findings was assessed by using the weighted kappa (κ) statistic. Between-group comparisons of subjects’ characteristics were performed using the Student t test. Parameters that were not normally distributed were analysed by Wilcoxon-Mann-Whitney test. Comparisons of categorical variables between groups were performed using the Chi2 or Fisher’s exact tests as appropriate. HRCT or BAL features associated with emphysema severity were determined by multiple regression analysis. All reported p values were two-sided, with a significance level set at p<0.05. Statistical analysis was performed using SAS version 9.4 (SAS Institute Inc., Cary, North Carolina, USA).

## Results

### Patient characteristics

**Fig 1:**
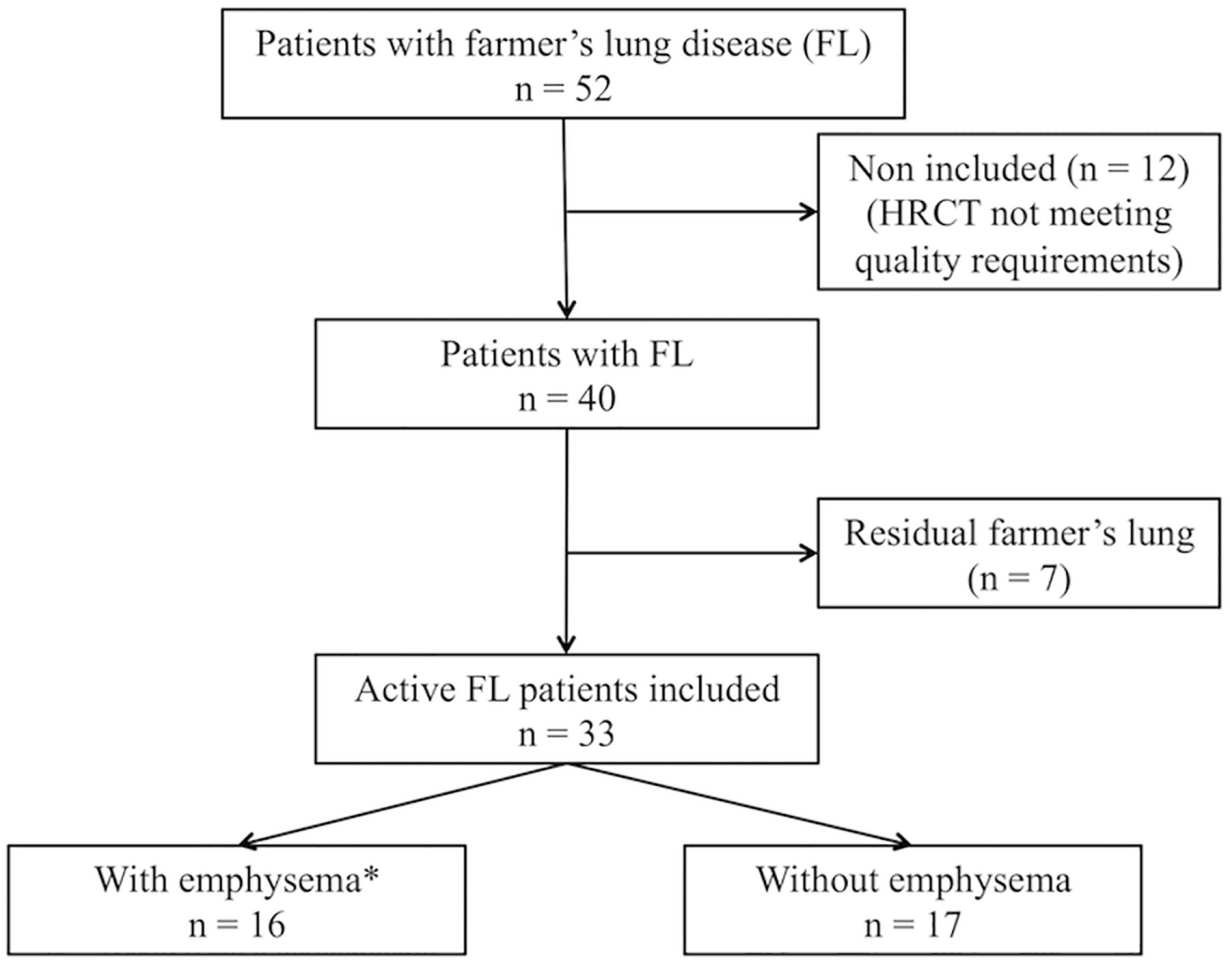
Flow chart of patients included in the study. * bilateral emphysema in at least three lobes.

Among 52 patients with HP who were pre-included in the SOPHIA study, 33 met the inclusion criteria of active FL and were included in the present study (Fig 1). The patient characteristics are presented in Table 1.

**Table 1:** Characteristics, respiratory symptoms and physical signs in patients with active farmer's lung disease.

	All active FL patients n = 33	Active FL patients with emphysema n = 16	Active FL patients without emphysema n = 17	p value
**Demographics**				
Male, n	24/73%	13/81%	11/65%	NS
Age, year	53.7±12.6	57.4±11.5	50.1±12.8	NS
Body mass index, kg/m^2^	24.7±5.2	24.7±5.1	24.7±5.6	NS
Tobacco, pack years	1.7±4.8	2.8±6.3	0.6±2.4	NS
Current/ex smoker, n	0/4	0/3	0/1	NS
**Clinical symptoms/history**				
Exposure to antigens	33/100%	16/100%	17/100%	NS
Still exposed	33/100%	16/100%	17/100%	NS
Duration of exposure, years	28.0±12.4	33.3±11.1	23.2±11.7	< 0.05
Median duration of symptoms, months	30.4±28.2	41.6±32.3	20.5±20.2	NS
Dyspnoea	30/91%	15/94%	15/88%	NS
mMRC scale (0 to 4)	2.1±0.9	2.2±0.8	2.1±1.0	NS
Cough	24/73%	9/56%	15/88%	NS
Sputum	15/45%	9/56%	6/35%	NS
Chills	16/48%	5/31%	11/65%	NS
Asthenia	17/52%	7/44%	10/59%	NS
Tightness of chest	4/12%	2/13%	2/12%	NS
Weight loss	10/30%	2/13%	8/47%	NS
Body aches	1/3%	1/6%	0/0%	NS
Wheezing	1/3%	1/6%	0/0%	NS
Chest (pleuritic) pain	3/9%	1/6%	2/12%	NS
Symptoms 4–8 h after exposure	14/42%	8/50%	6/35%	NS
Recurrent episodes of symptoms	26/79%	13/81%	13/76%	NS
**Physical signs**				
Fever	4/12%	0/0%	4/24%	NS
Inspiratory crackles	16/48%	6/38%	10/59%	NS
Wheezing	1/3%	1/6%	0/0%	NS
Cyanosis	1/3%	1/6%	0/0%	NS
Digital clubbing	0/0%	0/0%	0/0%	NS
Supraclavicular or cervical adenopathies	1/3%	0/0%	1/6%	NS
Heart failure	2/6%	1/6%	1/6%	NS

FL, farmer’s lung; mMRC = modified medical research council.

Values are means ± SD or number of patients / percentage of total.

p values are between active FL patients with emphysema vs. active FL patients without emphysema.

Bilateral emphysema in at least three lobes was found in 16 patients (48.5%) and fibrosis was identified in 4 patients (12%). Among those with emphysema, 12 did not present lung hyperinflation, including 2 cases with an association of emphysema and fibrosis.

Four patients (12%) had a history of smoking, but none of them were current smokers and no significant difference in tobacco smoking was found between patients with and without emphysema. Both groups had similar sex distribution, age, and body mass index (BMI). None of the patients were taking corticosteroids.

At the time of the present examination, all the patients were still exposed. Nevertheless, those with emphysema had a significantly longer duration of exposure compared to those without emphysema (p < 0.05). Recurrent episodes of FL and clinical symptoms, were not related to the presence of emphysema.

### HRCT

Interobserver agreement in the assessment of the CT findings was 85% (κ = 0.67). Ground glass opacities (GGO) and bronchial wall thickness (WT) were the most predominant findings in all cases (n = 26 and n = 24, respectively) (Table 2 and S1 Table). The prevalence of these patterns was similar between groups (n = 12 and n = 14 for GGO, n = 12 and n = 12 for WT, respectively in FL patients with and without emphysema). Mosaic perfusion and air trapping were less frequent in patients with emphysema compared to those without emphysema (n = 7 and n = 15 respectively for mosaic perfusion; p < 0.05). In 4 patients, a combination of honey combing pattern, reticulation and traction bronchiectasis made it possible to define the presence of fibrosis. Among these 4 patients, two had concomitant emphysema. None of the remaining 29 patients displayed any of these 3 features.

**Table 2:** Distribution and extent of small airways CT findings.

		Patients with emphysema n = 16		Patients without emphysema n = 17	
	Distribution	n	extent	n	extent
**Mosaic**	upper	6	1.3	14	1.7
	middle	7	1.2	14	1.7
	lower	7	1.1	15	1.9
	right	7	1.1	15	1.6
	left	7	1.1	14	1.9
**Air** trapping*	upper	4	1.3	10	2.1
	middle	5	1.1	10	1.9
	lower	6	1.0	10	2.0
	right	5	1.1	10	2.0
	left	6	0.9	10	2.0
**Emphysema**	upper	16	1.8		
	middle	15	1.6		
	lower	16	1.3		
	right	16	1.6		
	left	16	1.5		

Values are number of patients and mean extent scale.

Extent scale: (0: no feature; 1: 1–25%; 2: 26–50%; 3:51–75%; 4: more than 75%).

* data available for 20 patients.

**Fig 2:**
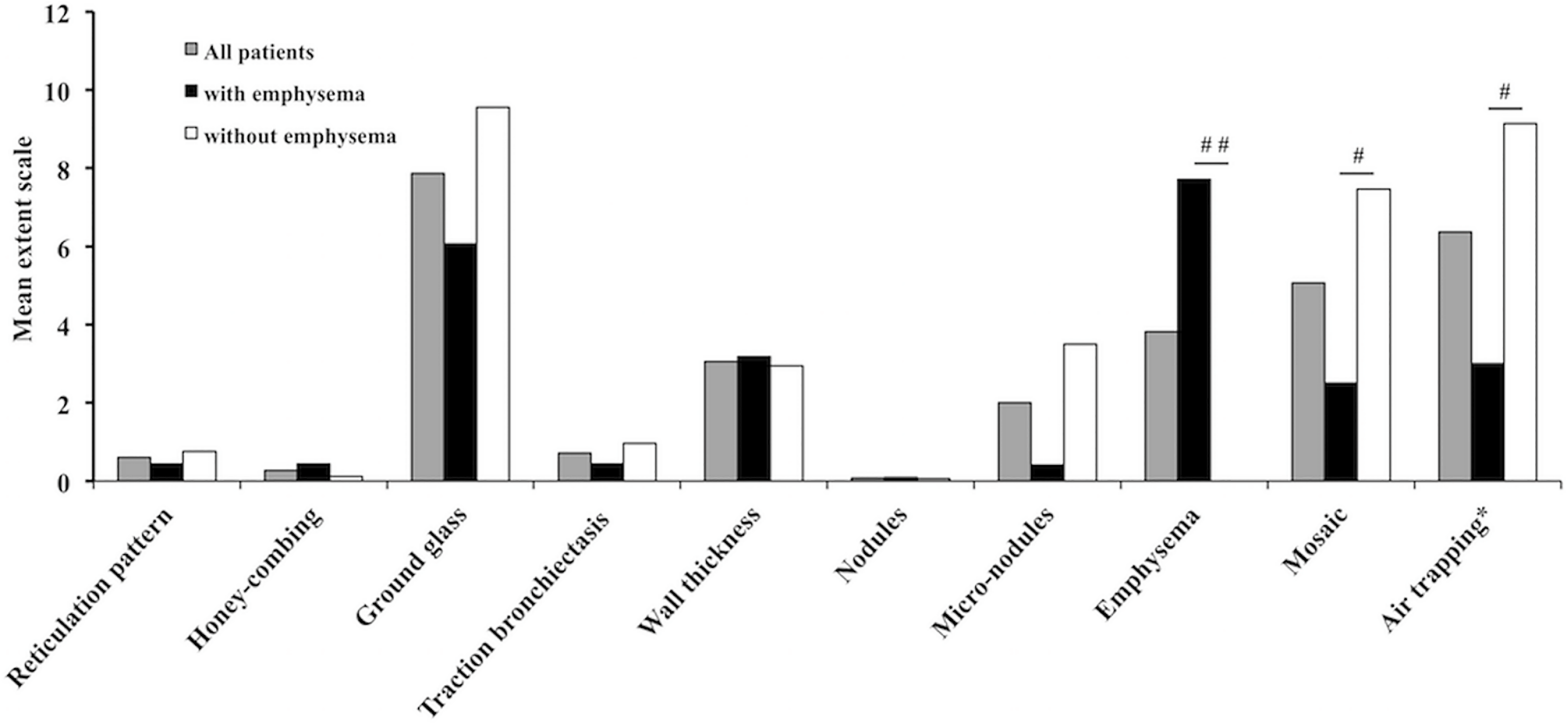
HRCT findings in patients with active FL. Mean of the individual sum of the extent scale for each group. The lungs of each patient were evaluated by lobe. In each lobe, a score of 0 to 4 was given for each of the features, therefore the maximal possible score was 20. ^#^ p < 0.01 and ^##^ p < 0.0001; * data available for 20 patients.

Among the 16 patients with emphysema, 15 displayed this feature in all lobes and 14 had a LAA over 5%. In addition, LAA was significantly higher and inspiratory density was lower in patients with emphysema compared to those without (respectively 14.54 ± 9.63 vs 4.26 ± 4.72; p < 0.001 and -820.7 ± 86.0 vs -763.7 ± 75.4; p < 0.01). Upper zone predominance and centrolobular distribution was found in most patients. Mean extent score of each feature is given in Fig 2. In addition, the severity of emphysema was inversely correlated to the extent of mosaic (r = -0.57) and of air trapping (r = -0.55).

### Pulmonary function and six minute walk test

The proportion of patients with airway obstruction defined by a forced expiratory volume in 1 s (FEV1)/forced vital capacity (FVC) ratio below the lower limit of normal (LLN) was significantly higher in patients with emphysema than in those without emphysema (n = 6 and n = 1, respectively; p < 0.05) (Table 3). Significant lung hyperinflation was found in 4 FL patients with emphysema (25%) and in one without emphysema. The transfer factor of the lung for carbon monoxide (TLCO) was not different between patients with and without emphysema (n = 10 and n = 7, respectively). Exercise-induced hypoxia was similar in both groups (n = 8 and n = 10, respectively in FL patient with and without emphysema). However, exercise-induced dyspnoea was greater in patients with emphysema compared to those without (n = 7 and n = 2, respectively; p < 0.05).

**Table 3:** Pulmonary function, laboratory blood work and broncho-alveolar lavage in patient with active FL.

	All active FL patients n = 33	Active FL patients with emphysema n = 16	Active FL patients without emphysema n = 17	p value
**Pulmonary function tests (preBD)**				
FEV_1_, L (z-score)	2.69±0.90 (-1.47±1.06)	2.51±0.78 (-1.69±1.15)	2.86±1.00 (-1.26±0.97)	NS
FEV_1_/ FVC, % (z-score)	0.75±0.12 (-0.48±1.54)	0.70±0.14 (-1.09±1.78)	0.80±0.07 (0.09±1.03)	< 0.05
TLC, L (z-score)	6.21±1.66 (-0.08±1.52)	6.74±1.64 (0.40±1.47)	5.60±1.51 (-0.64±1.44)	NS
FRC, L (z-score)	3.53±1.04 (0.50±1.47)	3.87±1.12 (0.89±1.59)	3.14±0.81 (0.04±1.24)	NS
RV, L (z-score)	2.41±0.84 (0.71±1.91)	2.80±0.86 (1.38±1.99)	1.95±0.58 (-0.07±1.53)	< 0.05
RV/TLC, L (z-score)	38.8±8.3 (0.49±1.38)	41.4±7.2 (0.79±1.41)	35.7±8.8 (0.15±1.32)	NS
TLCO, mL/min/mmHg (z-score)	19.0±8.3 (-2.17±1.65)	17.4±7.5(-2.43±1.81)	21.0±9.1 (-1.84±1.43)	NS
KCO, mL/min/mmHg/L (z-score)	3.3±1.1 (-1.00±1.12)	2.9±1.0(-1.34±1.28)	3.7±1.0 (-0.54±0.68)	NS
**6MWT**				
Distance, % predicted	96.2±13.8	97.2±13.8	95.3±14.3	NS
SpO2, delta	5.6±4.8	5.7±5.2	5.6±4.6	NS
Borg at end exercise	5.3±2.2	6.2±1.9	4.5±2.2	< 0.05
**Laboratory blood tests**				
PO_2_, kPa	8.55±1.07	8.53±1.09	8.58±1.15	NS
White blood cell count, 10^9^/L	7.6±2.8	6.4±2.0	8.2±3.0	NS
Lymphocytes, 10^9^/L	1.9±0.5	1.7±0.7	1.9±0.5	NS
Eosinophils, 10^9^/L	0.3±0.2	0.2±0.2	0.3±0.3	NS
**Broncho-alveolar lavage**				
Total cell count, 10^9^/L	485.2±338.8	480.0±376.0	490.0±311.3	NS
Lymphocytes, %	48±19	43±15	53±21	NS
Macrophages, %	39±19	46±18	31±18	< 0.05
Neutrophils, %	12±16	10±10	14±21	NS
Eosinophils, %	2±2	2±2	2±2	NS

*Definition of abbreviations*: BD = bronchodilator; FEV_1_ = forced expiratory volume in 1 second; FVC = forced vital capacity; TLC = total lung capacity; FRC = functional residual capacity; RV = residual volume; TLCO = diffusing capacity of the lung for carbon monoxide; KCO = carbon monoxide transfer coefficient; NS = not significantly different.

Values are means ± SD.

p values are between active FL patients with emphysema vs. active FL patients without emphysema.

### Precipitins tests and BAL

Disease activity was similar in both groups of FL patients as highlighted by comparable lymphocytic alveolitis (Table 3). There was no correlation between pulmonary functions and the BAL findings both for lymphocytes and mast cells. Nevertheless, lymphocytosis correlated with emphysema severity in the group of FL patients with emphysema (r = -0.49; p < 0.05) and in all patients (r = -0.37; p < 0.05).

Serum precipitins were not related to the presence of emphysema. However, two actinomyces species (mesophilic *Streptomyces* and *T*. *vulgaris*) were more frequently found in FL patients without emphysema (S2 Table).

### Occupational exposure

Among the 25 patients with complete occupational survey, those who had emphysema tended to have longer daily exposure and lived more often on traditional farms compared to those without emphysema. Indeed, the proportion of patients with emphysema (Table 4) was larger in those who used a traditional storage system compared to those who used modern storage techniques.

**Table 4:** Occupational characteristics of patients with active FL*.

	Active FL patients with emphysema	Active FL patients without emphysema	p-value
**Ever lived on a farm, n**	9/73%	7/50%	NS
**Farm characteristics, n**			
Separation between house and cowshed	6/55%	6/43%	NS
Central corridor	8/64%	8/57%	NS
Loose housing system	3/18%	9/64%	NS
Ventilation	4/36%	5/36%	NS
Traditional storage system	8/64%	3/21%	< 0.05
Farm modernization	7/55%	9/64%	NS
**Size of the farm, mean ± SD**			
Total size, hectares	62.5±12.2	88.5±41.5	NS
Size of fodder lands, hectares	37.2±14.6	60.4±29.4	< 0.05
Number of cows	36.3±13.5	47.8±31.1 5/36%	NS
**Current fodder handling, n**	5/45%		NS
**Hours spent each day in the farm, mean ± SD**	6.7±2.2	4.6±0.9	< 0.05

Values are means ± SD or number of patients / percentage of total.

*Data only available for 25 patients.

## Discussion

The main findings of this study are as follows: (i) the prevalence of emphysema in this incident active FL case series (48%) was largely higher than that of fibrosis; (ii) emphysema was mainly centrilobular and located in the upper lobes; most patients with emphysema did not have lung hyperinflation; (iii) the possible risk factors for emphysema in active FL were a longer duration of exposure to organic dusts and at a higher level.

It is commonly admitted that fibrosis is the most frequent finding in chronic and residual forms of HP. In contrast, there are few data regarding the prevalence of emphysema in FL. Twenty years ago, Erkinjuntti-Pekkanen et al. found that emphysema was a more frequent finding than interstitial fibrosis in FL [7]. Cormier et al. confirmed that emphysema was a direct consequence of FL, with emphysema being found in around 20% of never-smokers [8] with FL. Our study highlights that the prevalence of emphysema in FL may have been under-estimated. To exclude any interpretation bias, the findings of the radiologists were confirmed by automatic quantification: LAA over 5% was found in 14 out of 16 patients with emphysema.

In the two previous studies, the prevalence of emphysema was higher in ever smokers (ex or current smokers) than in never-smokers [7, 8]. In contrast, the proportion of ever-smokers among FL patients with emphysema was lower in our study compared to previous studies. It is therefore likely that tobacco smoking did not explain the very high prevalence of emphysema in our study.

Different exposure patterns could be partially responsible. In the previously mentioned studies, no distinction was made between active and residual forms, which were considered to be part of the same disease. In our study, we only included active FL. By design, we were therefore unable to study the association between disease activity level and risk of emphysema. Nevertheless, lymphocytic alveolitis is thought to represent an ongoing disease process rather than a normal immune response [8, 28]. Since the duration of exposure was higher in patients with active FL with emphysema, the persistence of exposure over time, and thus, lymphocytic alveolitis, could be a potential risk factor for emphysema. In addition, higher exposure levels were found in patients with emphysema. Indeed, our team previously reported that traditional fodder storage is associated with higher exposure levels to fungal and bacterial microorganisms [29]. It is therefore plausible that combined long-term and high level exposure are risk factors for emphysema in active FL.

Erkinjuntti-Pekkanen et al reported that recurrent attacks of FL were a risk factor for emphysema in FL [7]. We did not observe this association. Nevertheless, disease activity was not assessed in the study by Erkinjuntti-Pekkanen et al; and we therefore cannot exclude the possibility that FL patients who had recurrent episodes where also those who had active disease.

The present study showed that emphysema is a largely more prevalent finding than fibrosis in FL [6–8]. Cormier et al. demonstrated that mosaic and ground glass were the most prevalent finding firstly in patients with acute FL and secondly, in those with a history of FL still in contact with airborne contaminants. In contrast, these authors found that emphysema prevalence was similar in all groups, irrespective of the history and the current characteristics of FL. In our study, there was a balance between the usual signs of HP that reflect distal airway impairment, such as mosaic and trapping, and emphysema. Indeed, while emphysema and ground glass coexisted, the more emphysema present, the less mosaic observed. Although the relationships between bronchiolitis and emphysema remains largely unknown, this feature could support bronchiolitis as a driver of destructive emphysema. Another possible explanation may be that mosaic could be hidden by the presence of emphysema. Unfortunately, pulmonary function testing did not provide any further details. Indeed, a decrease in TLCO is a non-specific measurement of pulmonary alteration that can reflect parenchymal destruction (due to emphysema), heterogeneity of ventilation/perfusion (due to parenchymal inflammation) and/or lung hyperinflation (due to bronchiolitis and/or to emphysema).

Our results confirm that FL patients with emphysema have impaired TLCO and airway obstruction [7]. An original facet of this study was the absence of lung hyperinflation at rest in 75% of patients with extended emphysema. This is an unexpected result, as emphysema is associated with a decrease in pulmonary elastic recoil, and thus with static lung hyperinflation. Our findings suggest that in these patients, elastic recoil forces were abnormally increased in “healthy” non-emphysematous pulmonary parenchyma, leading to global normal lung elasticity, a finding that has previously been reported in patients with emphysema associated with lung fibrosis [30]. Elastin and collagen are two major components of the extracellular matrix, accounting for lung tissue viscoelastic mechanical properties. The most widely accepted hypothesis for tissue destruction in emphysema is the impairment of elastin [31]. Recent studies have also demonstrated that abnormalities in the collagen matrix could play a role [32]. Even cigarette smoke (chronic obstructive pulmonary disease) and immune-allergical reactions (HP) both involve chronic lung inflammation, pathophysiological processes leading to emphysema may be different involving diverse collagen-elastin network impairment. However, this needs to be histologically confirmed.

Finally among our 33 cases of active FL, 2 could be considered to have combined pulmonary fibrosis and emphysema [33]. Indeed, these two patients presented dyspnoea and inspiratory crackles on examination, emphysema in the upper lobes and fibrosis in the lower lobes on HRCT, while functional examination showed normal lung volume and spirometry.

### Limitations

The first limitation of our study was the absence of control groups with healthy exposed subjects. Indeed, the presence of emphysema could not only be the result of the immuno-allergic reaction of FL, but also of the chronic exposure to organic dust. Nevertheless, Hoppin et al. recently reported that the prevalence of emphysema in farmers was around 1.5% [34]. Thus, farming alone cannot explain the prevalence of emphysema detected in active FL.

The second limitation was the small population of FL patients in our study. Nevertheless, in Franche-Comté, the region where all the patients came from, the population of dairy farmers was estimated to be around 4000 between 2007 and 2015 [35]. Therefore the prevalence of FL in dairy farmers in our study (0.7%) was within the range of previously reported prevalence [1].

## Conclusion

In conclusion, our prospective study demonstrated that among patients with FL in which the disease remains active over time, around half develop emphysema. The fact that high level and long term exposure are risk factors of emphysema strengthens the need for early cessation of exposure or at least modernisation of farms with a view to preventing and reducing respiratory diseases in dairy farmers.

## Supporting information

S1 Table Distribution and extent of CT findings. (DOCX)

S2 Table Precipitins in patient with active FL. (DOCX)
