# Supplementary material for: Emphysema in active farmer’s lung disease
Source: PLoS One. 2017 Jun 14;12(6):e0178263. doi: 10.1371/journal.pone.0178263 (PMC5470683; doi:10.1371/journal.pone.0178263)
Supplement: S1 Table — (DOCX) [file pone.0178263.s001.docx]

| **Supplementary Table 1.** Distribution and extent of CT findings | | | | | |
| --- | --- | --- | --- | --- | --- |
|  |  | Patients  with emphysema  n = 16 | | Patients  without emphysema n = 17 | |
|  |  |  |  |  |  |
|  |  |  |  |  |  |
|  | Distribution | n | extent | n | extent |
| **Ground  glass** | upper | 9 | 2.0 | 13 | 2.4 |
|  | middle | 9 | 2.1 | 13 | 2.5 |
|  | lower | 12 | 1.8 | 14 | 2.5 |
|  | right | 12 | 1.6 | 14 | 2.3 |
|  | left | 12 | 1.6 | 14 | 2.3 |
| **Honey combing** | upper | 1 | 1.0 | 1 | 0.8 |
|  | middle | 1 | 1.0 | 0 |  |
|  | lower | 2 | 1.0 | 1 | 0.3 |
|  | right | 2 | 0.7 | 2 | 0.3 |
|  | left | 2 | 0.8 | 1 | 0.3 |
| **Traction bronchiectasis** | upper | 1 | 1.0 | 2 | 1.8 |
|  | middle | 1 | 1.0 | 2 | 1.8 |
|  | lower | 2 | 1.0 | 2 | 1.5 |
|  | right | 2 | 0.7 | 2 | 1.6 |
|  | left | 2 | 0.8 | 2 | 1.8 |
| **Reticulation** | upper | 1 | 1.0 | 1 | 2.3 |
|  | middle | 1 | 1.0 | 1 | 2.5 |
|  | lower | 2 | 1.0 | 2 | 1.5 |
|  | right | 2 | 0.7 | 2 | 1.3 |
|  | left | 2 | 0.8 | 2 | 1.3 |
| **Wall  thickness** | upper | 12 | 0.9 | 12 | 0.8 |
|  | middle | 11 | 0.9 | 12 | 0.8 |
|  | lower | 11 | 0.9 | 12 | 0.8 |
|  | right | 12 | 0.8 | 12 | 0.8 |
|  | left | 12 | 0.9 | 12 | 0.8 |
| **Nodules** | upper | 0 |  | 0 |  |
|  | middle | 1 | 1 | 0 |  |
|  | lower | 1 | 0.3 | 1 | 0.5 |
|  | right | 1 | 0.3 | 1 | 0.3 |
|  | left | 1 | 0.3 | 0 |  |
| **Micro nodules** | upper | 1 | 2.3 | 4 | 3.1 |
|  | middle | 0 |  | 4 | 3.1 |
|  | lower | 1 | 1.0 | 5 | 2.3 |
|  | right | 1 | 0.5 | 5 | 2.4 |
|  | left | 1 | 2.5 | 4 | 2.9 |

Values are number of patients and mean extent scale

Extent scale: (0: no feature; 1: 1-25%; 2: 26-50%; 3:51-75%; 4: more than 75%)
