# Supplementary material for: Emphysema in active farmer’s lung disease
Source: PLoS One. 2017 Jun 14;12(6):e0178263. doi: 10.1371/journal.pone.0178263 (PMC5470683; doi:10.1371/journal.pone.0178263)
Supplement: S2 Table — (DOCX) [file pone.0178263.s002.docx]

| **Supplementary Table 2.** Precipitins in patient with active FL | | | | | | | | | | | |  |  |
| --- | --- | --- | --- | --- | --- | --- | --- | --- | --- | --- | --- | --- | --- |
|  | All active FL patients n = 33 | | |  | Active FL patients  with emphysema n = 16 | | |  | Active FL patients  without emphysema n = 17 | | |  |  |
|  |  |  |  |  |  |  |  |  |  |  |  |  | p-value |
|  |  |  |  |  |  |  |  |  |  |  |  |  |  |
| **Positive precipitins to known antigens, n / %** | 29 | / | 94% |  | 14 | / | 93% |  | 15 | / | 94% |  | NS |
| **Fungal antigens** | 27 | / | 87% |  | 13 | / | 87% |  | 14 | / | 88% |  | NS |
| Lichtheimia corymbifera | 11 | / | 35% |  | 5 | / | 33% |  | 6 | / | 38% |  | NS |
| Wallemia sebi | 21 | / | 68% |  | 11 | / | 73% |  | 10 | / | 63% |  | NS |
| Eurotium amstelodami | 10 | / | 32% |  | 3 | / | 20% |  | 7 | / | 44% |  | NS |
| Fusarium oxysporum | 9 | / | 29% |  | 6 | / | 40% |  | 3 | / | 19% |  | NS |
|  |  |  |  |  |  |  |  |  |  |  |  |  |  |
| **Bacterial antigens** | 16 | / | 52% |  | 6 | / | 40% |  | 10 | / | 63% |  | NS |
| Saccharopolyspora rectivirgula | 13 | / | 42% |  | 6 | / | 40% |  | 7 | / | 44% |  | NS |
| Thermoactinomyces vulgaris | 10 | / | 32% |  | 1 | / | 7% |  | 9 | / | 56% |  | < 0.01 |
| Streptomyces mesophile | 6 | / | 19% |  | 0 |  |  |  | 6 | / | 38% |  | < 0.05 |
| Saccharomonospora viridis | 9 | / | 29% |  | 5 | / | 33% |  | 4 | / | 25% |  | NS |
|  |  |  |  |  |  |  |  |  |  |  |  |  |  |

Values are number of patients / percentage of total.

p values are between active FL patients with emphysema vs active FL patients without emphysema.
